# Early Identification of Autism Spectrum Disorder (ASD): Strategies for Use in Local Communities

**DOI:** 10.1007/s12098-022-04172-6

**Published:** 2022-05-23

**Authors:** Roula Choueiri, William T. Garrison, Valerie Tokatli

**Affiliations:** 1 Autism Spectrum Center, Department of Neurology, Boston Children’s Hospital, 2 Brookline Place, Brookline, MA 02445 USA; 2 Division of Developmental and Behavioral Pediatrics, University of Massachusetts Chan Medical School, Worcester, MA USA

**Keywords:** Autism spectrum disorder (ASD), Early identification, Screening for ASD, Low- and middle-income countries and ASD, Low-cost ASD screening, Cultural factors and ASD, Community workers, Community early childhood providers

## Abstract

Early diagnosis of autism spectrum disorder (ASD) is essential for improved outcomes. There is a paucity of data on the prevalence of ASD in low- and middle-income countries (LMIC), but early identification may be further delayed in those communities. In this paper, recent studies on strategies for the early detection of ASD, and the prevalence of ASD in LMIC are reviewed. The limitations that can arise in the early identification of ASD in LMIC communities are discussed, and screening tools and strategies that can be helpful are identified. The goal is to recommend models that are culturally appropriate and scientifically valid, easily integrated within community settings while strengthening community systems and reducing disparities in the early identification of ASD. Starting locally by simplifying and demystifying the ASD identification process and building community connections will inform global researchers and policymakers while making a difference in the lives of the children and families affected by ASD.

## Introduction

Autism spectrum disorder (ASD) includes a group of neurodevelopmental disorders that present with delayed social communication skills and restricted repetitive behaviors [1]. The assessment of ASD remains clinical, and its early identification and interventions are crucial in improving outcomes [2]. The American Academy of Pediatrics [3] continues to reinforce autism screening in primary care. But even in the US, when the age of parental concern can be as early as 12–18 mo, the diagnosis of ASD continues to be made closer to 4 y of age, and those from different ethnic backgrounds and low socioeconomic status are even further delayed [4]. There is a paucity of community-based data on developmental status and disability in young children residing in low- and middle-income countries (LMIC), despite the fact that most children with disabilities live in these countries [5, 6]. This review paper, though not exhaustive, identifies important studies completed in LMIC on ASD prevalence, screening, and diagnosis. The authors also seek to present information on various validated tools and implementation strategies that have been shown to be effective in low-resource community settings to improve the early identification of ASD.

## Definitions of Income Countries

The World Bank [7] assigns the world’s economies to four income groups—low, lower-middle, upper-middle, and high-income countries. The classifications are based on gross national income per capita in current US dollars (USD) from the previous year. For the year 2021, the classifications are:

**Table:** 

**Group**	
Low income	< 1,046
Lower-middle income	1,046–4,095
Upper-middle income	4,096–12,695
High income	> 12,695

## Current Strategies for Early Identification of ASD

In addition to promoting and reinforcing autism screening in primary care for children aged 18 to 36 mo, which continues to be challenging, other strategies to improve the early detection of ASD have been developed. A review of 40 studies [8] noted several promising programs. Such programs emphasize specialized ASD training of primary care providers, completion of developmental screening in the emergency room for all children 18 mo old and increasing general public awareness about the early signs of developmental delay and ASD. However, this review showed findings to be mixed, and no reliable tracking of outcomes noted. In addition, the ADOS-2 (Autism Diagnostic Observation Schedule) [9] has been the gold standard tool for diagnosis of ASD, but it is a labor-intensive and prohibitively expensive observational system to use widely in LMIC and even in underserved areas in high-income countries. Tools and models exploring other valid ways to provide a diagnosis in those most vulnerable without access to resources, have been developed. In addition to standardized checklists and structured interviews, new strategies have included observational and child-interactive screenings with level-2 screening measures such as the RITA-T (Rapid Interactive Screening Test for Autism in Toddlers) [10, 11] and the STAT (Screening Test for Autism in Toddlers) [12, 13]*.* Implementation models incorporating level-2 tools have shown improved triaging of referrals, increased and faster access, and provided diagnosis for those in the moderate to severe range without prolonged diagnostic evaluations [14, 15]. In addition, to bolster identification of high-risk children, training the community early childhood providers such as early intervention (EI) staff on those screening measures has improved identification and access in a diverse and underserved community [16]. Another strategy has been gaining momentum—the Autism Extension for Community Healthcare Outcomes (ECHO Autism) model [17]. This approach connects primary care clinicians from remote areas to autism specialists through ongoing education and mentoring. The overarching goal of this effort is to build capacity and clinical skills for the early identification of ASD in addition to appropriate management of children with ASD. A relatively new approach not specific to ASD but to any developmental, behavioral, and emotional condition, has emerged—the International Interprofessional Collaborative Office Rounds (iiCOR) [18], which consists of multidisciplinary groups meeting regularly to discuss cases from participating practices and includes teams from North America, Africa, Asia, and South America. In addition, telehealth evaluations have emerged from the uniquely pressing necessity created by the COVID-19 pandemic and have also expanded possibilities for new remote video tools for the screening and diagnosis of ASD. However, these methods are dramatically affected by socioeconomic and geographical disparities [19].

## Prevalence of ASD in LMIC

Studies from Colombia, India, Jamaica, Jordan, and Mexico [20] show that the parental concerns about child development increase around 21–22 mo of age, and the eventual diagnosis of ASD is likely to occur at a mean age between 45 and 57 mo. Earlier concern was noted for boys, with higher educated parents, or if there is a physical issue. A newer study from Brazil also highlights the same lag difference [21] but describes further the negative experience parents, mostly mothers, felt while seeking diagnostic clarification.

The global incidence of ASD has been increasing progressively and is now estimated to be in the 1% range [22] but incidence in LMIC is unknown. In contrast, the prevalence of ASD in the US has an average of 16.8 per 1000 children over the age of 8 y [4]. A study from Kolkata, India [23], showed an estimated weighted prevalence of ASD in school-age children to be 0.23%, which the authors estimated to represent the lower limit of true prevalence. A recent review of 12 studies looking at the prevalence of ASD in Asia [24] showed that the general prevalence is 0.36% with males (0.45%) more affected than females (0.18%), and that the prevalence was highest in East Asia (0.50%). However, the heterogeneity in age, setting, and tools in the different studies was underlined. In a rural community in Bangladesh, a study in children aged 18–36 mo showed a prevalence of 0.75/1000, whereas the prevalence of cerebral palsy and of developmental delay were 5.6/1000 and 2.6/1000, respectively [25].

To put this in perspective, the mortality rate has decreased between 1990 and 2016 for those under 5 y, globally, from 11 to 5 million, respectively. However, the rates of developmental disabilities remain similar: 52.9 million (8.4%) children worldwide were found to have at least 1 of 6 developmental disabilities (ADHD, epilepsy, ASD, hearing loss, vision loss, intellectual disability) in 2016, as compared to 53.0 million (8.9%) in 1990. An estimated 95% of those children live in LMIC. So, although mortality rates have improved, there do not seem to be adequate measures to identify those who are surviving and are at increased risk for developing neurodevelopmental disabilities, such as ASD [6].

## Screening and Diagnosis of ASD in LMIC

There are many challenges to identifying an appropriate screening and/or diagnostic test for ASD that can be feasible within an LMIC community. Such a measure must be culturally and linguistically valid. In addition, an important barrier to implementation of screening and diagnostic tools for ASD is the fact that many tools are copyrighted and require permissions and payment for translation into other languages [5]. Other important factors to consider when thinking about screening and/or diagnostic tests in LMIC include:

*Literacy levels:* In Africa, literacy levels range from 30 to 90% [26]. In India, literacy levels vary between 61 and 91% depending on the state [27]. In addition, even with those with adequate literacy, filling forms may not be a common cultural concept but rather interpersonal communication [28].

*Culture:* There is a dearth of literature on cultural perception of developmental disabilities and of ASD [29]. In addition, a clear understanding of what developmental milestones are, and what is considered a delay may vary between communities and countries [30]. Carruthers et al. showed core signs of ASD to be similar in children 4–9y from India, UK, and Japan, but also found cultural variations [31]. However, others [32] found that ASD has different social constructs across countries and cultures. Stigma, *karma*, guilt, can affect parental perceptions, as well as willingness to seek diagnosis and treatment for ASD. With current political and population shifts [33], additional complicating factors include migration, prolonged residence in a refugee camp, or a community that mistrusts healthcare workers.

Further obvious barriers are limited access to trained diagnosticians, the financial cost, length of training, complexity of tool administration, and available expertise in these countries to continuously train new providers to use these tools. For example, Marlow et al. recommends tools that community workers can train and administer easily [34]. Families usually have an ongoing trusting relationship with their community workers (early intervention, daycare, preschool);thus, parents may be successfully engaged in screening even more easily than in healthcare settings [16].

### Current Tools Available for the Screening and Diagnosis of ASD in Children Younger than 3 Years

Table 1 compiles a list of useful screening and diagnostic tests for those younger than 3 y. This is not an exhaustive list. The authors list when available, costs and translations, as well as sensitivity and specificity of each test, and reported positive predictive value (PPV). Screening tests can be divided into level-1 and level-2, and they can be either questionnaires or interactive tests. Those are important elements to consider when thinking about literacy in parents, culture, and age of the child.

**Table 1:** Screening and diagnostic measures for those under 3 y

Screening tool	Age			Format	Time to complete in minutes		Sensitivity/Specificity		Availability		Cost			Translated to other languages?
*Level 1*														
MCHAT-R/F	16–36 mo			Questionnaire: 20 items Follow-up questionnaire, if score 3–7	5–10		At >= 2 0.94/0.83 and PPV: 98% DD and 54% ASD		www.Mchatscreen.com		Free to download			Yes: Translations in 75 languages
CSBSDP-IT	6–24 mo			Up to 24 questions that assess communication	5–10		Scores at age 1 y associated with ASD at age 3 y		https://brookespublishing.com/wp-content/uploads/2012/06/csbs-dp-itc.pdf		Free to download			No
*Level 2 interactive*														
STAT	24–36 mo			Interactive, training needed	20–30		0.92/0.85 (for severe autism)		Purchase: VU e-innovations		STAT Test Kit: $500			No
RITA-T	18–36 mo			Interactive, training needed	10		1/0.84 (all ages)		www.childrenshospital.org/autismRITA-T		RITA-T Online Training & Kit: $260			Yes: Spanish, Portuguese, French, Arabic In process: Turkish and Hindi
*Diagnostic tool*														
ADOS- 2	12 mo–adulthood			Interactive, behavior and observation, training needed	30–60		90.9%/66.0%		Purchase: Western Psychological Services		ADOS-2 Kit: $2,395			Yes: Translations in 9 languages
ADI-R	2 y & up			Interview, training needed	90–150		0.67%–1.00%/0.64%–94%		Purchase: Western Psychological Services		ADI-R Kit: $320			Yes: Translations in 16 languages
CARS-2	2 y & up			Observation 15 items	15–20		Agreement with DSM-5: 84%		Purchase: Western Psychological Services		CARS-2 Kit: $237			Yes: Translations in Bulgarian and Italian
TASI	12–36 mo			Interview, observation and history - 37 interview questions	N/A		52.83%/92.72%		https://mchatscreen.com/tasi/		Free to download			No
*Telehealth measures*														
Tele-RITA-T [46]	18–36 mo			Telehealth interactive, training required	10		High correlation with RITA-T at score more than 9 for ASD		www.childrenshospital.org/autismRITA-T		Free to download with the RITA-T training			Yes: Translations in Spanish and Portuguese
Tele-ASD-PEDS [47]	14–36 mo			Telehealth interactive, training needed	10–20		N/A		https://vkc.vumc.org/vkc/triad/tele-asd-peds		Free to download			No
NODA [48]	18 mo–6y			Observation, training needed	40		84.9%/85.7%		https://www.nodaautismdiagnosis.com/		$500			No
BOSA [49]	Minimally verbal age & up			Observation, interactive, training needed	45–60		N/A		Used in conjunction with the ADOS-2: https://www.semel.ucla.edu/autism/bosa-training		Free BOSA material along with ADOS-2 Kit			No

Screening tools: *CSBSDP-IT* Communication and Symbolic Behavior Scales Development Profile, Infant–Toddler Checklist, *MCHAT-R/F* Modified Checklist for Autism in Toddlers–Revised with Follow-Up, *RITA-T* Rapid Interactive Screening Test for Autism in Toddlers, *STAT* Screening Tool for Autism in Toddlers and Young Children

Diagnostic: *ADI-R* Autism Diagnostic Interview, *ADOS-2* Autism Diagnostic Observation, Second Edition, *CARS-2* Childhood Autism Rating Scale, Second Edition, *TASI* Toddler Autism Symptom Interview

Telehealth measures: *BOSA * Brief Observation of Symptoms of Autism, *NODA * Naturalistic Observational Diagnostic Assessment, *Tele-ASD-PEDS * Telemedicine-Based ASD Evaluation Tool for Toddlers and Young Children, *Tele-RITA-T * Televideo Rapid Interactive Screening Tool for Autism in Toddlers

A level-1 screening test is a screening measure administered to a well-child population to tease out those at risk for developmental and/or ASD concerns. The best example for a level-1 test is the MCHAT-R/F (Modified Checklist for Autism in Toddlers–Revised with Follow-up Interview) [35] which is used widely. The MCHAT-R/F is administered as a questionnaire for parents to complete and has been translated into many languages. Administering the MCHAT-R/F yields close to 98%–99% PPV for developmental delay but only 54% for ASD. In addition, a large percentage of children who never receive the MCHAT-R/F will also be missed in community screening. Starting from a group of children defined at risk, such as from EI, such identification improved to 60%–78% [36]. Furthermore, the identification of ASD from the MCHAT-R/F improved much more when the providers in EI asked the questions to families as interview, instead of giving them the questionnaire to complete [16]. Similarly, Li et al. found that administering the CHAT-23 (Checklist for Autism in Toddlers) as an interview with the family and administering the follow-up interview improved early detection in a community-based model in Shanghai [22]. Using the CSBSDP-IT (Communication and Symbolic Behavior Scales Development Profile, Infant–Toddler Checklist) as a level-1 measure is helpful in those younger than 18 mo. Although not an ASD screener, its score at 12 mo of age was associated with ASD at 3 y of age [37].

Level-2 screening tools, are tests that are administered to a group already identified as at risk for ASD. They can be questionnaires or interactive; however, in young children aged 18 mo to 3 y, it is recommended to have an interactive system, (i.e., a play-based test that the child can participate in and that can elicit early signs for ASD). For this age group, there are two major interactive measures: the STAT [12, 13] and the RITA-T [10, 14]. The STAT has different cutoff scores for those younger than 24 mo: a cutoff score at 2.75 shows specificity of 0.73 and positive predictive value of 0.56 [12]. For those 24–36 mo, a cutoff score of 2 yields 0.92 sensitivity and 0.85 specificity. However, it can miss milder forms of ASD [14] which comprise a large portion of the rising case incidence in most countries. The STAT takes 20–30 min to administer and score, and has an extensive training-to-reliability process, in addition to costs that would be prohibitive in many settings. It has not been translated or validated in non-English languages.

The RITA-T has specificity of 1 and PPV of 1 at a score of 16 and above, meaning that it is picking up with great reliability those with ASD. Even in the medium-risk range, or a score between 12 and 16, the RITA-T offers a specificity of 83%–96% and PPV of 93%–98%, which is still significantly strong [16]. Its administration and scoring occur within 10 min and reliable training can be achieved in 3–4 h. A variety of community early childhood providers have trained on the RITA-T, including social workers, speech and language and occupational therapists, nurses, physician assistants, and pediatricians. Cutoff scores have been consistent across published studies for those 18 mo–36 mo. A recent study showed that using RITA-T for those older than 3 y, can still be helpful and correlated with ASD diagnosis, in addition to its similar performance in other ethnic groups [38]. The RITA-T does not rely on language; its manual and scoring sheet are translated into several languages [39] and its validation in other cultures is in process. It is a low-cost test, and easy to assemble with only 7 elements in the kit. In addition, the authors are working on a RITA-T training module to be available in the public domain.

A two-level screening model [40] integrates a level-1 screening with a level-2 measure to identify those at risk for developmental delay, and further, those at a high risk for ASD.

**Fig. 1:**
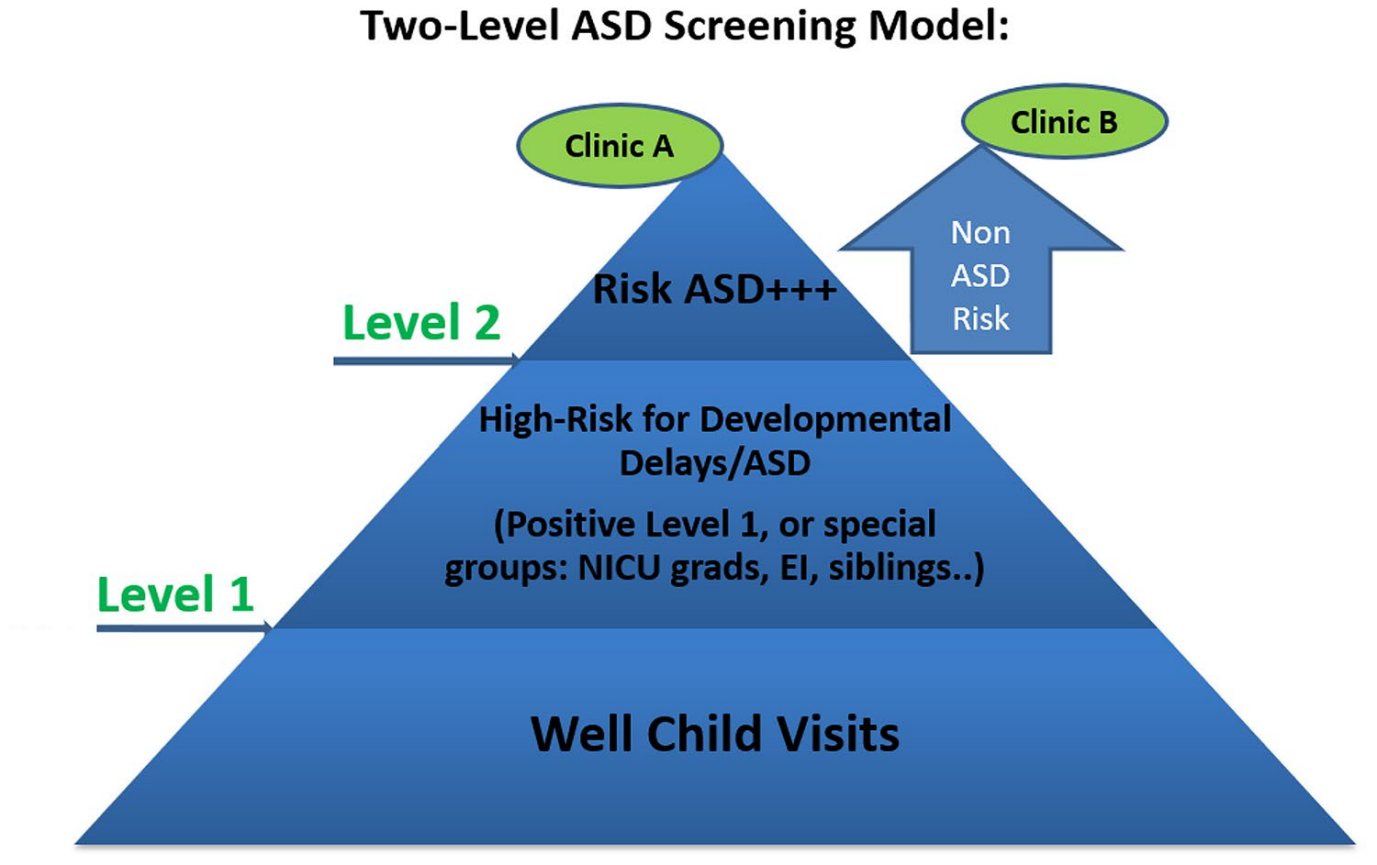
Two-level ASD screening model. Reprinted from Improving Early Identification and Access to Diagnosis of Autism Spectrum Disorder in Toddlers in a Culturally Diverse Community with the Rapid Interactive Screening Test for Autism in Toddlers, by R. Choueiri, A. Lindbaum, M. Ravi, W. Robsky, J. Flahive, and W. Garrison, 2021, Journal of Autism and Developmental Disorders*,* 51, p. 3938. Copyright 2021 The Author(s). *ASD* Autism spectrum disorder, *EI* Early intervention staff, *NICU* Neonatal Intensive Care Unit

This approach will help to identify those at elevated risk for ASD (Fig. 1). Marlow et al. [34] identified various screening tools for ASD developed specifically for LMIC/non-Western settings. Table 2 lists those tools. Each of those measures can be integrated into this model.

**Table 2:** Screening tools for ASD, developed for LMIC/non-Western settings

	Screening tool	References	Used to screen for	Used in	Age range (months/years)	Rater (R)/Observation (O)	No. of items/Length of test	Sensitivity/Specificity above 70	Sample > 300	Free	Used in LMIC
23Q	23-item screener	Kakooza-Mwesige et al. [50]	NDD	Uganda	2–9 y	R	23 items	√	√	√	√
HIVA	HIVA	Samadi and McConkey [51, 52]	ASD	Iran	3–11 y	R	10 items	√√	√	√	√
INCLEN-ASD	INCLEN Diagnostic Tool for Assessment of Autism	Juneja et al. [53]	ASD	India	2–9 y	R+O	41 items, 45–60 min	√√		√*	√
ISAA	Indian Scale for Assessment of Autism	Mukherjee, Malhotra, Aneja, Chakraborty, and Deshpande [54]; Patra and Arun [55]	ASD	India	3–22 y	O	40 items, 15–20 min	√√	√	√	√
PAAS	Pictorial Autism Assessment Schedule	Perera et al. [56]; Perera, Jeewandara, Seneviratne, and Guruge [57]	ASD	Sri Lanka	18–48 mo	R	21 items	√√		√*	√
TIDOS	Three-Item Direct Observation Scale	Oner, Oner, and Munir [58]		Turkey	18–60 mo	R+O	3 items (O) 40 items (R)	√√		√*	√

Tools that appear to be free (i.e., no purchase cost involved or tool described as low-cost), received a checkmark with an asterisk (√*). Tools received a checkmark with an asterisk (√*) if the tool was designed for a non-Western setting or aboriginal populations within in a HIC. * Screening tools for ASD, developed for LMIC/non-Western settings. Reprinted from “A Review of Screening Tools for the Identification of Autism Spectrum Disorders and Developmental Delay in Infants and Young Children: Recommendations for Use in Low- and Middle-Income Countries” by M. Marlow, C. Servili, M. Tomlinson, 2019, Autism Research: The official journal of the International Society for Autism Research*, 51,* p. 186.Copyright 2019 The Authors. Autism Research published by International Society for Autism Research published by Wiley Periodicals, Inc.

Diagnostic assessment comprises parent-generated information as well as structured and validated observational tools. Questionnaires include the ADI-R (Autism Diagnostic Interview–Revised) [41], and more recently, the TASI (Toddler Autism Symptom Interview) [42]. Diagnostic tools can be observational, such as the CARS-2 (Childhood Autism Rating Scale) [43] or interactive such as the ADOS-2 [9]. All measures rely on DSM-5 criteria for the diagnosis of ASD [1]. For the purpose of this paper, developing telehealth measures will not be reviewed in detail, but they have been listed in Table 1.

## Strategies

Here, the information and practical recommendations to improve early ASD identification in LMIC are provided.

A Healthcare/Community Worker Setting

**Fig. 2:**
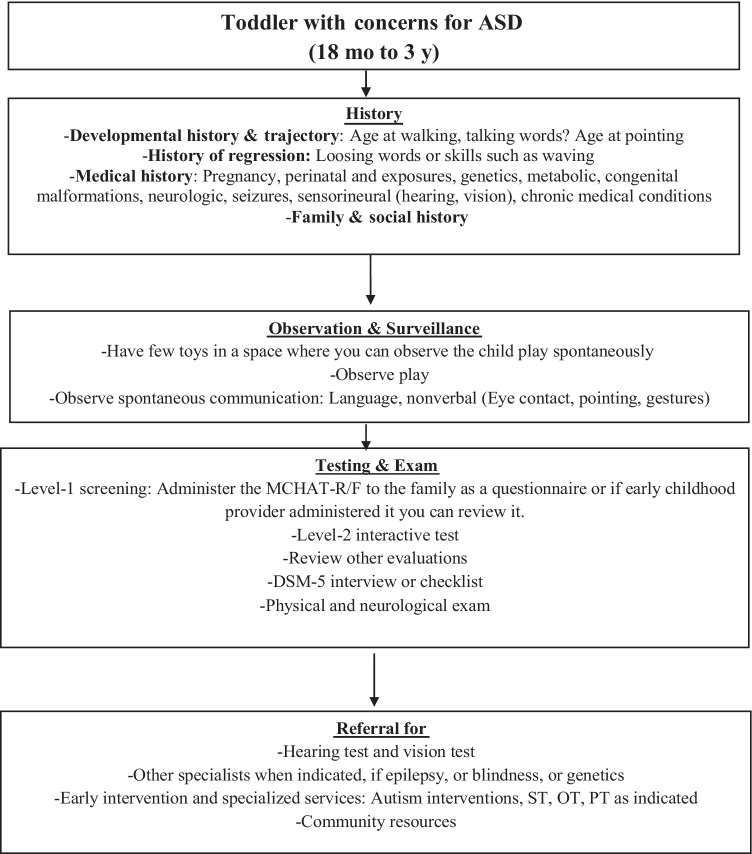
ASD assessment. Adapted from “New Assessments and Treatments in ASD,” by R. Choueiri and A. Zimmerman, 2017, Current treatment options in Neurology,19:6p. 9. Copyright 2017 by Springer Science + Business Media. *ASD* Autism spectrum disorder, *DSM-5* Diagnostic and Statistical Manual of Mental Disorders, Fifth Edition, *OT* Occupational therapy, *PT* Physical therapy, *ST* Speech therapy

1. It is important to always start with a good history that includes pregnancy and medical history, as well as milestones age acquisition such as walking or first words. A history of regression, whether language or social is always important to obtain and can represent first signs of ASD [44].
2. Observation of spontaneous play—observing how the child interacts with culturally appropriate and available toys—can provide significant information; repetitive play, lining up objects, spinning, high interest toys, and sensory seeking behaviors are strong observational elements and part of the DSM-5 for the diagnosis of ASD.
3. Based on previous studies, and observations, it is best to administer a level-1 test, such as the CSBSDP-IT or the MCHAT-R/F as an interview and use the follow-up interview subsequently as recommended [35].
4. Applying a two-level screening model identifies those with great risk of ASD. A level-2 screener that is interactive is better at eliciting behaviors delayed in those 18–36 mo old. In addition, it provides an opportunity to educate parents on the skills that are delayed and that are consistent with ASD symptoms.
5. The level-2 screening test has to be easy to train and integrate in community settings and validated in other languages and cultures.
6. Adding a DSM-5 checklist should then be sufficient in a large number of toddlers with a question of ASD. Using CARS-2 can be helpful, if available. Figure 2 incorporates these recommendations.

B. Community Settings and Beyond

1. It is important to know the community systems, stakeholders, and early childhood providers. Becoming familiar with resources, autism and disability centers, preschools, and intervention programs is key to developing those connections that are essential for improved identification.
2. Partnering with a tertiary care center, if and when this is available, is beneficial. A good example is the model reported by Lemay et al. [14]. They applied a two-level screening model to referrals received by their community and divided toddlers into three groups: low, medium, and high risk. Only those in the medium-risk category received the full-length ASD evaluation. Those in the high-risk group were provided the ASD diagnosis after administration of the DSM-5 checklist. This model led to dramatically improved access.
3. Partnering with community workers/early childhood providers—training early childhood providers on the early signs of ASD and on the RITA-T was associated with improved access [16]. This is a preferred strategy for low-resource communities. Families usually have an ongoing relationship with their community early childhood providers. Hence, they can be engaged more easily in screening and in discussion of ASD concerns. The community provider can then administer screening, and prepare the family for a diagnosis. This will create further support for the family and readiness to move forward with planning for interventions.
4. ECHO Autism model: Successful results about the integration and adaptation of this strategy in LMIC are starting to be shared [45].
5. Increasing awareness about early signs of ASD and/or developmental delay and demystifying ASD is important. The more it is out there, the less scary it is.
6. Finding and adapting material about developmental expectations and concerns to share with the families. Material has to be adapted to the current community and culture [29].
7. It is key to translate and validate screening and diagnostic tests in different cultural groups and to have these tests be open access.
8. Identification of an academic center or a nongovernmental organization partner in a community or county/region, or those interested to partner, and to fund such initiatives.

## Conclusions

​The current state of the early identification of ASD globally has been reviewed in this paper. The authors also sought to provide practical recommendations and strategies to providers and public health planners in a range of LMIC. While there is a need to understand better the prevalence and presentation of ASD in LMIC, the literature demonstrates the importance to work locally within communities on building awareness, capacity, and clinical skills to identify the early signs of ASD. It is essential to find culturally appropriate ways to approach concerns of ASD with families, and to provide screening with tools that are validated, low cost or free, and easy to train and integrate in different settings. To be successful, any such models must collaborate with community-based early childhood workers.

## References

[1] American Psychiatric Association. Diagnostic and statistical manual of mental disorders: DSM-5 (5th ed.). Arlington; VA, American Psychiatric Association. 2013. p.50–9.

[2] Landa RJ (2018). Efficacy of early interventions for infants and young children with, and at risk for, autism spectrum disorders. Int Rev Psychiatry.

[3] Hyman SL, Levy SE, Myers SM. Council on Children with Disabilities, Section on Developmental and Behavioral Pediatrics. Identification, evaluation, and management of children with autism spectrum disorder. Pediatrics. 2020;145:e20193447. 10.1542/peds.2019-3447 31843864

[4] Baio J, Wiggins L, Christensen DL (2018). Prevalence of autism spectrum disorder among children aged 8 years – autism and developmental disabilities monitoring network, 11 sites, United States, 2014. MMWR Surveill Summ.

[5] Durkin MS, Elsabbagh M, Barbaro J (2015). Autism screening and diagnosis in low resource settings: Challenges and opportunities to enhance research and services worldwide. Autism Res.

[6] Global Research on Developmental Disabilities Collaborators (2018). Developmental disabilities among children younger than 5 years in 195 countries and territories, 1990–2016: a systematic analysis for the Global Burden of Disease Study 2016. Lancet Glob Health.

[7] New World Bank country classifications by income level: 2020–2021. World Bank Blogs. 2020. Available at: https://blogs.worldbank.org/opendata/new-world-bank-country-classifications-income-level-2020-2021. Accessed on 13 Oct 2021.

[8] Daniels AM, Halladay AK, Shih A, Elder LM, Dawson G (2014). Approaches to enhancing the early detection of autism spectrum disorders: a systematic review of the literature. J Am Acad Child Adolesc Psychiatry.

[9] Lord C, Rutter M, DiLavore PC, Risi S, Gotham K, Bishop S. Autism diagnostic observation schedule (2nd ed.). Torrance, CA: Western Psychological Services. 2012.

[10] Choueiri R, Wagner S (2015). A new interactive screening test for autism spectrum disorders in toddlers. J Pediatr.

[11] RITA–T – Rapid interactive screening test for autism in toddlers. Boston Children's Hospital. 2022. Available at: www.childrenshospital.org/autismRITA-T. Accessed on 12 Apr 2022.

[12] Stone WL, McMahon CR, Henderson LM (2008). Use of the screening tool for autism in two-year-olds (STAT) for children under 24 months: an exploratory study: An exploratory study. Autism.

[13] Stone WL, Coonrod EE, Turner LM, Pozdol SL (2004). Psychometric properties of the STAT for early autism screening. J Autism Dev Disord.

[14] Lemay JF, Amin P, Langenberger S, McLeod S (2020). Experience with the rapid interactive test for autism in Toddlers in an autism spectrum disorder diagnostic clinic. J Dev Behav Pediatr.

[15] Mazurek MO, Curran A, Burnette C, Sohl K (2019). ECHO autism STAT: accelerating early access to autism diagnosis. J Autism Dev Disord.

[16] Choueiri R, Lindenbaum A, Ravi M, Robsky W, Flahive J, Garrison W (2021). Improving early identification and access to diagnosis of autism spectrum disorder in toddlers in a culturally diverse community with the rapid interactive screening test for autism in toddlers. J Autism Dev Disord.

[17] Sohl K, Mazurek MO, Brown R (2017). ECHO autism: using technology and mentorship to bridge gaps, increase access to care, and bring best practice autism care to primary care. Clin Pediatr (Phila).

[18] Kiing JSH, Feldman HM, Ladish C, et al. International interprofessional collaborative office rounds (iiCOR): addressing children’s developmental, behavioral, and emotional health using distance technology. Front Public Health. 2021;9:657780. 10.3389/fpubh.2021.657780 PMC8149584 34055722

[19] Amaral DG, de Vries PJ (2020). COVID-19 and autism research: perspectives from around the globe. Autism Res.

[20] Samms-Vaughan ME (2014). The status of early identification and early intervention in autism spectrum disorders in lower- and middle-income countries. Int J Speech Lang Pathol.

[21] Gomes PT, Lima LH, Bueno MK, Araújo LA, Souza NM (2015). Autism in Brazil: a systematic review of family challenges and coping strategies. J Pediatr (Rio J).

[22] Li C, Zhu G, Feng J (2018). Improving the early screening procedure for autism spectrum disorder in young children: experience from a community-based model in Shanghai. Autism Res.

[23] Rudra A, Belmonte MK, Soni PK, Banerjee S, Mukerji S, Chakrabarti B (2017). Prevalence of autism spectrum disorder and autistic symptoms in a school-based cohort of children in Kolkata. India Autism Res.

[24] Qiu S, Lu Y, Li Y, et al. Prevalence of autism spectrum disorder in Asia: a systematic review and meta-analysis. Psychiatry Res. 2020;284:112679. 10.1016/j.psychres.2019.112679 31735373

[25] Akhter S, Hussain AHME, Shefa J, Kundu GK, Rahman F, Biswas A. Prevalence of autism spectrum disorder (ASD) among the children aged 18–36 months in a rural community of Bangladesh: a cross sectional study. F1000Res. 2018;7:424. 10.12688/f1000research.13563.1 PMC6039957 30026928

[26] De Vries PJ (2016). Thinking globally to meet local needs: autism spectrum disorders in Africa and other low-resource environments. Curr Opin Neurol.

[27] Literacy rate of India 2021. Census of India 2021. Available at: https://censusofindia2021.com/literacy-rate-of-india-2021/. Accessed on 13 Oct 2021.

[28] Berg A (2012). Connecting with South Africa: Cultural communication and understanding.

[29] Samadi SA (2020). Parental beliefs and feelings about autism spectrum disorder in Iran. Int J Environ Res Public Health.

[30] Ertem IO, Krishnamurthy V, Mulaudzi MC (2018). Similarities and differences in child development from birth to age 3 years by sex and across four countries: a cross-sectional, observational study. Lancet Glob Health.

[31] Carruthers S, Kinnaird E, Rudra A (2018). A cross-cultural study of autistic traits across India, Japan, and the UK. Mol Autism.

[32] Kim HU (2012). Autism across cultures: rethinking autism. Disabil Soc.

[33] Kroening AL, Moore JA, Welch TR, Halterman JS, Hyman SL. Developmental screening of refugees: a qualitative study. Pediatrics. 2016;138:e20160234. 10.1542/peds.2016-0234 PMC5005020 27527798

[34] Marlow M, Servili C, Tomlinson M (2019). A review of screening tools for the identification of autism spectrum disorders and developmental delay in infants and young children: recommendations for use in low- and middle-income countries. Autism Res.

[35] Robins DL, Casagrande K, Barton M, Chen CM, Dumont-Mathieu T, Fein D (2014). Validation of the modified checklist for autism in toddlers, revised with follow-up (M-CHAT-R/F). Pediatrics.

[36] Pandey J, Verbalis A, Robins DL (2008). Screening for autism in older and younger toddlers with the modified checklist for autism in toddlers. Autism.

[37] Pierce K, Carter C, Weinfeld M (2011). Detecting, studying, and treating autism early: the one-year well-baby check-up approach. J Pediatr.

[38] Kong XJ, Sherman HT, Tian R, et al. Validation of rapid interactive screening Test for autism in toddlers using autism diagnostic observation schedule^TM^ second edition in children at high-risk for autism spectrum disorder. Front Psych. 2021;12:737890. . 10.3389/fpsyt.2021.737890 PMC8517472 34658971

[39] Yassin R, Abou Abbas L, Krayem M, Salame E, Choueiri R, Boustany R-M (2020). The rapid interactive screening test for autism in toddlers (RITA-T): validity in a Lebanese cross-cultural pilot study. Int J Autism & Relat Disabil.

[40] Oosterling IJ, Wensing M, Swinkels SH (2010). Advancing early detection of autism spectrum disorder by applying an integrated two-stage screening approach. J Child Psychol Psychiatry.

[41] Kim SH, Thurm A, Shumway S, Lord C (2013). Multisite study of new autism diagnostic interview-revised (ADI-R) algorithms for toddlers and young preschoolers. J Autism Dev Disord.

[42] Coulter KL, Barton ML, Boorstein H (2021). The toddler autism symptom inventory: use in diagnostic evaluations of toddlers. Autism.

[43] Moulton E, Bradbury K, Barton M, Fein D (2019). Factor analysis of the childhood autism rating scale in a sample of two-year olds with an autism spectrum disorder. J Autism Dev Disord.

[44] Filipek PA, Accardo PJ, Ashwal S (2000). Practice parameter: screening and diagnosis of autism: report of the quality standards subcommittee of the American academy of neurology and the child neurology society. Neurology.

[45] Giachetto G, Casuriaga AL, Santoro A, et al. Extension for community healthcare outcomes Uruguay: a new strategy to promote best primary care practice for autism. Glob Pediatr Health. 2019;6:2333794X19833734. 10.1177/2333794X19833734 PMC6446250 31044151

[46] Choueiri R, Garrison W, Tokatli V, Ravi M, Prashad E. Screening for autism with the telehealth Rapid Interactive Screening Test for Autism in Toddlers (RITA-T). Available at: https://virtual2021.pas-meeting.org/2021/PAS/fsPopup.asp?efp=WldIRlFRV1gxNDAzOA&PosterID=367603&rnd=0.8299802&mode=posterinfo. Accessed on 12 Apr 2022.

[47] Corona, L., Hine, J., Nicholson, A., Stone, C., Swanson, A., Wade, J., Wagner, L., Weitlauf, A., & Warren, Z. TELE-ASD-PEDS: A Telemedicine-based ASD Evaluation Tool for Toddlers and Young Children. Vanderbilt University Medical Center. 2020. Available at: https://vkc.vumc.org/vkc/triad/tele-asd-peds. Accessed on 13 Oct 2021.

[48] Smith CJ, Rozga A, Matthews N, Oberleitner R, Nazneen N, Abowd G. Investigating the accuracy of a novel telehealth diagnostic approach for autism spectrum disorder. Psychol Assess. 2017;29:245–52. 10.1037/pas0000317 PMC5116282 27196689

[49] Rynkiewicz A, Vasa R, Łucka I, Mazur A. Use of the Brief Observation of Symptoms of Autism (BOSA) as a new clinical approach to assessing patients with suspected spectrum disorder during the COVID-19 pandemic. Pediatr Pol. 2020;95:241–3.

[50] Kakooza-Mwesige A, Ssebyala K, Karamagi C, et al. Adaptation of the “ten questions” to screen for autism and other neurodevelopmental disorders in Uganda. Autism. 2014;18:447–57. 10.1177/1362361313475848 23536263

[51] Samadi SA, McConkey R. The utility of the gilliam autism rating scale for identifying Iranian children with autism. Disabil Rehabil. 2014;36:452–56. 10.3109/09638288.2013.797514 23738615

[52] Samadi SA, McConkey R. Screening for autism in Iranian preschoolers: Contrasting M-CHAT and a scale developed in Iran. J Autism Dev Disord. 2015;45:2908–16. 10.1007/s10803-015-2454-1 25911978

[53] Juneja M, Mishra D, Russel PS, et al. INCLEN diagnostic tool for autism spectrum disorder (INDT-ASD): Development and validation. Indian Pediatr. 2014;51:359–65. 10.1007/s13312-014-0417-9 24953575

[54] Mukherjee SB, Malhotra MK, Aneja S, Chakraborty S, Deshpande S. Diagnostic accuracy of indian scale for assessment of autism (ISAA) in children aged 2-9 years. Indian Pediatr. 2015;52:212–16. 10.1007/s13312-015-0608-z 25848996

[55] Patra S, Arun P. Use of Indian scale for assessment of autism in child guidance clinic: an experience. Indian J Psychol Med. 2011;33:217–19. 10.4103/0253-7176.92043 PMC3271508 22345858

[56] Perera H, Wijewardena K, Aluthwelage R. Screening of 18–24-month-old children for autism in a semi-urban community in Sri Lanka. J Trop Pediatr. 2009;55:402–5. 10.1093/tropej/fmp031 19401407

[57] Perera H, Jeewandara KC, Seneviratne S, Guruge C. Culturally adapted pictorial screening tool for autism spectrum disorder: A new approach. World J Clin Pediatr. 2017;8:45–51. 10.5409/wjcp.v6.i1.45 PMC5296629 28224095

[58] Oner P, Oner O, Munir K. Three-item direct observation screen (TIDOS) for autism spectrum disorder. Autism. 2014;18:733–42. 10.1177/1362361313487028 PMC3986348 24126869

